# METTL14 Acts as a Potential Regulator of Tumor Immune and Progression in Clear Cell Renal Cell Carcinoma

**DOI:** 10.3389/fgene.2021.609174

**Published:** 2021-05-28

**Authors:** Tianbo Xu, Su Gao, Hailong Ruan, Jingchong Liu, Yuenan Liu, Di Liu, Junwei Tong, Jian Shi, Hongmei Yang, Ke Chen, Xiaoping Zhang

**Affiliations:** ^1^Department of Urology, Union Hospital, Tongji Medical College, Huazhong University of Science and Technology, Wuhan, China; ^2^Department of Geriatrics, Union Hospital, Tongji Medical College, Huazhong University of Science and Technology, Wuhan, China; ^3^Institute of Gerontology, Union Hospital, Tongji Medical College, Huazhong University of Science and Technology, Wuhan, China; ^4^Department of Pathogenic Biology, School of Basic Medicine, Huazhong University of Science and Technology, Wuhan, China

**Keywords:** clear cell renal cell carcinoma, m6A RNA methylation, METTL14, CCL5, regulatory T cell, tumor immunity, tumor progression

## Abstract

Clear cell renal cell carcinoma (ccRCC) is characterized by its insensitivity to chemoradiotherapy and lacks effective diagnostic and prognostic biomarkers. In this study, we focused on the role of m6A RNA methylation regulators for tumor immunity. Based on the expression of 20 m6A regulators, consensus clustering was performed to divide patients into cluster1/cluster2 and showed that there was a survival difference between the two clusters. Through cox regression analysis, five hub m6A regulators were screened to construct a risk model. Further analysis showed that the risk score was an independent prognostic factor. GSEA, GSVA, and KEGG analysis revealed that immune cell pathways played a critical role between the high risk group and low risk group. Combined with CIBERSORT and survival analysis, five hub tumor-infiltrating immune cells (TIICs) were identified for further study. Meanwhile, correlation analysis indicated that IGF2BP2 was positively associated with activated memory CD4 T cell and METTL14 was negatively correlated to the regulatory T cell. Therefore, IGF2BP2 and METTL14 were regarded as key genes. Further study verified that only METTL14 possessed good diagnostic and prognostic value. Then, GSEA exhibited that METTL14 was mainly enriched in chemokine related pathways. We also found that CCL5 was negatively correlated to METTL14 and might serve as a potential target of METTL14. In conclusion, these findings suggest that the METTL14/CCL5/Tregs axis is a potential signaling pathway for regulating tumor immunity, and might become novel therapeutic targets for ccRCC.

## Introduction

Renal cell carcinoma (RCC) is one of the most common solid tumors in the urinary system. In the United States, the latest research infers that there will be 73,750 new cases and 14,830 people will die of this cancer in 2020 (Siegel et al., 2020). According to the survival data from the Surveillance, Epidemiology, and End Results (SEER) database, the 5-year relative survival is 75.2%^1^. Clear cell renal cell carcinoma (ccRCC) is the most frequent subtype of RCC, and accounts for 70–80% (Moch et al., 2016) of cases. Due to the insensitivity of chemoradiotherapy and hidden clinical manifestations, the mortality of ccRCC is persistently high and surgery is still the primary treatment (Ljungberg et al., 2015; Capitanio and Montorsi, 2016). Moreover, approximately 30% of patients have distant metastasis at the first time of diagnosis, which leads to the loss of the chance for surgery (Gong et al., 2016). It is, therefore, necessary to discover novel diagnostic and prognostic biomarkers and therapeutic strategies for ccRCC.

At present, many studies have verified that post-translational RNA modifications play a vital role in various diseases (Prieto et al., 2020), especially cancers (Huang et al., 2020). Among these modifications, m6A RNA methylation has been considered as the most pervasive modification in regulating RNA transcription (Barbieri et al., 2017), RNA splicing (Zhao et al., 2014), RNA degradation (Du et al., 2016), etc. It has been reported that m6A methylation was performed via a methyltransferase complex composed of “writer,” “eraser,” and “reader” (Chen et al., 2019; Lan et al., 2019). Methyltransferase like 14 (METTL14), a member of “writer,” are capable of catalyzing the formation of m6A. Previous studies have demonstrated that METTL14 was closely associated with tumorigenesis and progression. In bladder cancer, Gu et al. revealed that METTL14 could inhibit tumor initiating cells (TIC) self-renewal and tumorigenesis (Gu et al., 2019). It has been reported that METTL14 acts as a tumor suppressor for inhibiting tumor metastasis via regulating miRNA in hepatocellular carcinoma (Ma et al., 2016). Similarly, Yang et al. also verified that METTL14 suppressed the proliferation and metastasis of colorectal cancer through mediating lncRNA XIST (Yang et al., 2020). In breast cancer, METTL14 serves as an oncogene and drives tumor cell migration and invasion (Yi et al., 2020). However, the study of METTL14 is lacking and the molecular mechanisms are unclear in ccRCC.

In this study, we focused on the functions of m6A RNA methylation regulators and selected METTL14 as a key gene for in-deep study in ccRCC. Through comprehensive bioinformatics analysis, the correlation and potential mechanisms between METTL14 and tumor immune were further clarified.

## Materials and Methods

### Data Collection and Study Design

All gene expression data and clinical traits data were obtained from The Cancer Genome Atlas (TCGA) database^2^. The study design is shown in detail in the flow diagram Supplementary Figure 1.

### Identification of Differentially Expressed Genes (DEGs) or m6A Regulators (DE-m6A)

The “DESeq2” (Love et al., 2014) and “edgeR” (Robinson et al., 2009) packages were used to screen DEGs or DE-m6A with adjusted *p*-value <0.05 and | log2(FoldChange)| > 2. The wilcoxon test was utilized to identify differentially expressed m6A regulators according to a cutoff criterion of adjusted *p*-value <0.05.

### Correlation Analysis

The “cor.mtest” algorithm and “corrplot” package^3^ were used to perform spearman correlation analysis and visualize the results. *P*-value <0.05 was selected as a cutoff point.

### Construction of Protein-Protein Interaction (PPI) Network

The m6A RNA methylation regulators were submitted to the STRING database^4^ for constructing the PPI network. Moreover, cytoscape (Shannon et al., 2003) software was used to visualize and analyze the network.

### Consensus Clustering of m6A Regulators

The “ConsensusClusterPlus” package (Wilkerson and Hayes, 2010) was utilized to divide ccRCC patients into different clusters (k = 2–9). Moreover, we performed survival analysis to evaluate the prognostic value of different clusters. *P*-value <0.05 was considered statistically significant.

### Principal Component Analysis (PCA)

In this study, PCA was performed by using “limma” package (Ritchie et al., 2015) and visualized via using “ggplot2” package^5^ in R program.

### Construction of Cox Proportional Hazard Regression Model

The “survival” package^6^ was applied to perform a univariate cox regression analysis for preliminarily assessing the prognostic value of m6A regulators. Least Absolute Shrinkage and Selection Operator (LASSO) regression analysis was then used to further screen key prognostic factors and build a risk model via running “glmnet” (Friedman et al., 2010) and “survival” packages in R. The risk score (RS) formula as following:

$$RS=\sum_{i=1}^{n}Coef\left(i\right)X\left(i\right)$$

The Coef (i) represents the coefficient and the X(i) represents the expression of selected genes. Moreover, univariate and multivariate cox regression analyses were used to assess the prognostic value of the risk model.

### Survival Analysis and Receiver Operator Characteristic (ROC) Curve Analysis

According to the median of gene expression/risk score/TIICs abundance, ccRCC patients were divided into two groups (high group and low group). The “survival” package and GraphPad Prism software were utilized to draw the survival curves. A log rank *p*-value <0.05 was considered statistically significant. We performed further ROC curves to evaluate the diagnostic value.

### Gene Set Enrichment Analysis (GSEA) and Gene Set Variation Analysis (GSVA)

All ccRCC patients were divided into high group and low group. Then, GSEA online tool (Subramanian et al., 2005) was used to find potential molecular mechanisms. In addition, the “GSVA” package (Hänzelmann et al., 2013) was applied to screen differential signaling pathways between the high risk group and the low risk group.

### Kyoto Encyclopedia of Genes and Genomes (KEGG) Pathway Enrichment Analysis

To further analyze the biological function of DEGs, KEGG enrichment analysis was performed via running the “clusterProfiler” package (Yu et al., 2012) in the R program. *P*-value <0.05 was selected as the cutoff point.

### Purity Analysis of Stromal Cell and Immune Cell

The “estimate” package (Yoshihara et al., 2013) was applied to identify the purity of stromal cells and immune cells. Then, we performed a survival analysis to evaluate the prognostic value.

### Distribution of Tumor-Infiltrating Immune Cells (TIICs)

CIBERSORT algorithm^7^ with LM22 signature was utilized to calculate the relative abundance of 22 TIICs at 1,000 permutations. *P*-value <0.05 was considered statistically significant.

### Genetic Alteration Analysis and Immunohistochemistry (IHC) Analysis

The “cBioPortal” online tool (Cerami et al., 2012; Gao et al., 2013) was used to analyze the genetic alteration of hub m6A regulators. Furthermore, the IHC results of METTL14 were obtained from The Human Protein Atlas (HPA) database.

### Statistical Analysis

In this study, SPSS 22.0 and GraphPad Prism 7.0 software were applied to process data. All data were represented as mean ± SD. Student’s *t*-test and Chi-square test were used to analyze the data. *P*-value <0.05 was considered statistically significant.

## Results

### The Expression Level and Interaction of m6A RNA Methylation Regulators in ccRCC

**FIGURE 1:**
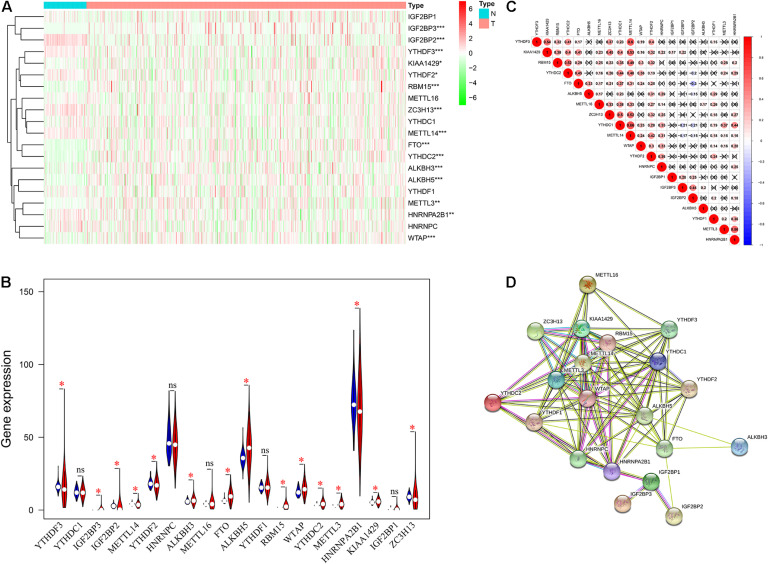
The expression level and interaction of twenty m6A RNA methylation regulators in ccRCC. **(A,B)** The expression level of m6A regulators between ccRCC tissues and normal tissues. **(C)** Spearman correlation among the 20 m6A regulators. **(D)** PPI network of m6A regulators following STRING database. ccRCC, clear cell renal cell carcinoma; PPI, protein-protein interaction. *P < 0.05; **P < 0.01; ***P < 0.001.

Gene expression data were obtained from the TCGA KIRC dataset. As shown in Figures 1A,B, a total of 15 differentially expressed m6A regulators (DE-m6A) were identified. There were six up-regulated m6A regulators (YTHDF3, IGF2BP2, METTL14, YTHDF2, HNRNPA2B1, and ZC3H13) and nine down-regulated m6A regulators (IGF2BP3, KIAA1429, RBM15, FTO, YTHDC2, ALKBH3, ALKBH5, METTL3, and WTAP). Moreover, correlation analysis indicated that METTL14 was likely to be the hub gene and strongly positively correlated to YTHDF3 (R = 0.60), KIAA1429 (R = 0.53), ZC3H13 (R = 0.52), and YTHDC1 (R = 0.66) (Figure 1C). Further PPI network analysis exhibited that all m6A regulators had close interactions with each other, especially METTL14 (Figure 1D).

### Consensus Clustering of m6A RNA Methylation Regulators

**FIGURE 2:**
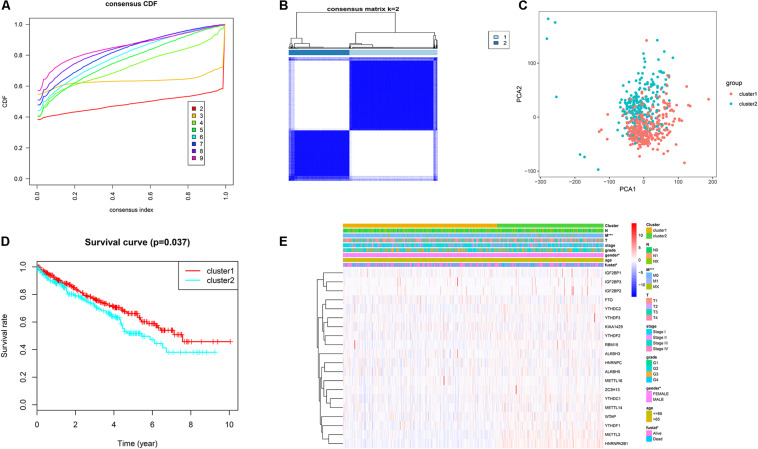
Consensus clustering of m6A regulators. **(A)** Cumulative distributive function (CDF) for k = 2–9. **(B)** ccRCC patients were divided into two clusters for k = 2. **(C)** PCA for total RNA expression pattern. Red represents cluster1 and green represents cluster2. **(D)** Patients within cluster1 had better OS. **(E)** The distinction of clinical traits between cluster1 and cluster2. ccRCC, clear cell renal cell carcinoma; PCA, principal component analysis; OS, overall survival. *P < 0.05; **P < 0.01; ***P < 0.001.

Based on the expression similarity of m6A regulators, k = 2 was selected as the threshold for dividing the patients into two subgroups (cluster1 and cluster2) in the TCGA KIRC dataset (Figures 2A,B). PCA further verified that there was an obvious difference in transcription profile between cluster1 and cluster2 (Figure 2C). Moreover, we found that patients in the cluster2 subgroup had poor overall survival (OS) (Figure 2D). Cox regression analysis showed that the cluster was an independent prognostic factor (Table 1). Combined with clinical characteristics, results revealed that M stage, gender, and survival state had a significant distinction between the above two subgroups (Figure 2E).

**TABLE 1:** The cox regression analysis of two clusters.

	Univariate			Multivariate		
	HR	95% CI	*P* value	HR	95% CI	*P* value
Age	1.03	1.02–1.05	0.00	1.04	1.02–1.05	0.00
Gender	0.96	0.69–1.32	0.79	1.10	0.78–1.55	0.57
Grade	2.33	1.88–2.88	0.00	1.56	1.23–1.98	0.00
Stage	1.94	1.69–2.22	0.00	1.79	1.22–2.63	0.00
T	1.99	1.68–2.36	0.00	0.84	0.59–1.21	0.35
N	3.56	1.88–6.77	0.00	1.79	0.91–3.50	0.09
M	4.41	3.20–6.07	0.00	1.14	0.63–2.05	0.67
Cluster	1.41	1.03–1.91	0.03	1.43	1.03–1.98	0.03

*HR, hazard rate; CI, confidence interval.*

### Cox Regression Analysis and Construction of Risk Model

**FIGURE 3:**
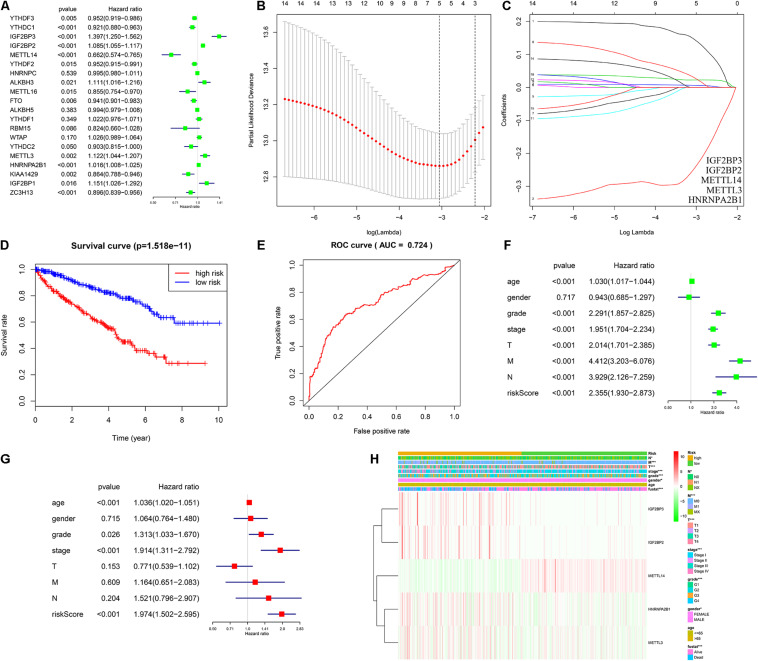
Construction of risk model and assessment of its diagnostic and prognostic value. **(A)** Univariate cox regression analysis of twenty m6A regulators. **(B,C)** Five genes (IGF2BP3, IGF2BP2, METTL14, METTL3, and HNRNPA2B1) were selected as the key variate for the risk model via LASSO cox regression. **(D)** Patients with high risk have a poor OS. **(E)** ROC curve showed that the risk model had a good diagnostic value (AUC = 0.724). **(F,G)** Univariate and multivariate cox regression verified that risk score was an independent prognostic factor. **(H)** The distinction of pathological characteristics between high risk group and low risk group. LASSO, least absolute shrinkage and selection operator; OS, overall survival; ROC, receiver operating characteristic; AUC, area under the curve. *P < 0.05; **P < 0.01; ***P < 0.001.

To evaluate the prognostic value of m6A regulators, univariate and LASSO cox regression analyses were performed. As shown in Figure 3A, a total of fourteen m6A regulators were regarded as prognostic factors (*p* < 0.05). In LASSO cox regression analysis, five hub m6A regulators (IGF2BP3, IGF2BP2, METTL14, METTL3, and HNRNPA2B1) were screened for the construction of a risk model (Figures 3B,C). Furthermore, the associated coefficients were obtained from LASSO analysis. The risk score = 0.147^∗^IGF2BP3 expression – 0.247^∗^METTL14 expression + 0.023^∗^IGF2BP2 expression + 0.006^∗^ HNRNPA2B1 expression + 0.030^∗^METTL3 expression. According to the risk score, the ccRCC patients were divided into a high risk group and a low risk group. Survival analysis indicated that patients with a high risk score had shorter overall survival times (Figure 3D). In addition, the ROC curve exhibited that the risk score had a good diagnostic value (Figure 3E, AUC = 0.724). Further univariate (Figure 3F, HR = 2.355, *p* < 0.001) and multivariate (Figure 3G) cox regression analysis demonstrated that risk score was a potential independent prognostic factor in ccRCC. Moreover, we also found that the risk score was significantly correlated to the T stage, N stage, M stage, TNM stage, grade, gender, and survival state (Figure 3H).

### The Risk Model Was Closely Associated With Immune-Related Pathways

**FIGURE 4:**
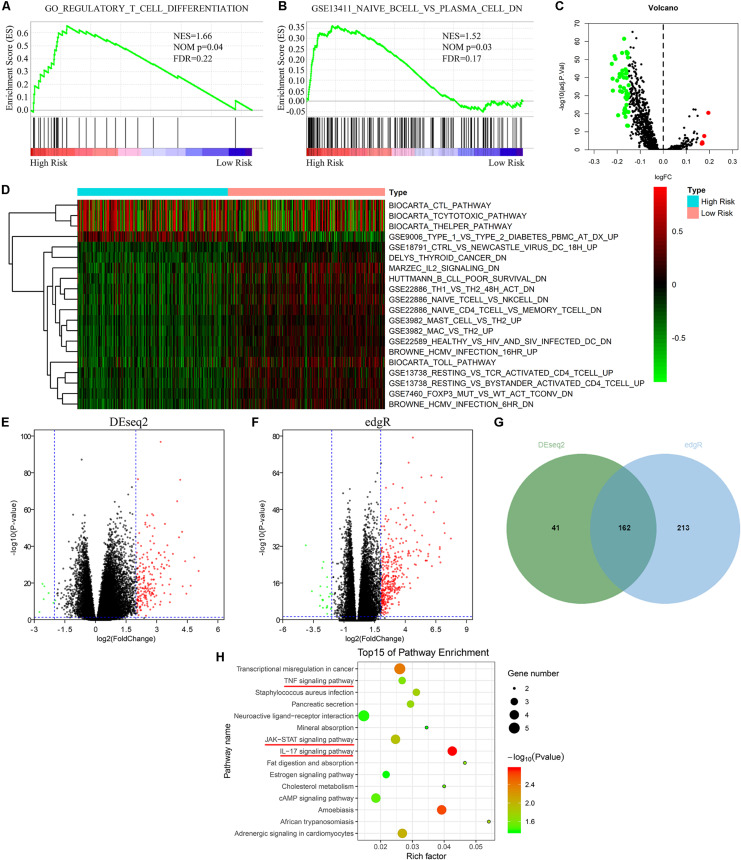
Risk signature was closely associated with immune-related pathways. **(A,B)** GSEA indicated that the high risk group was mainly enriched in immune cell related pathways. **(C,D)** GSVA showed that there were many differentially expressed immune cell-related pathways between the high risk group and the low risk group. **(E–G)** Identification of DEGs between high risk group and low risk group via “DESeq2” and “edgeR” packages. **(H)** KEGG pathway enrichment analysis of DEGs between high risk group and low risk group. GSEA, gene set enrichment analysis; GSVA, gene set variation analysis; DEGs, differentially expressed genes; KEGG, Kyoto Encyclopedia of Genes and Genomes.

To investigate potential molecular mechanisms of the risk model, GSEA, GSVA, and KEGG pathway analyses were performed. GSEA indicated that the high risk group was mainly enriched in immune cell-related pathways (Figures 4A,B). Further GSVA also verified that there were many differentially expressed immune cell-related pathways between the high risk group and the low risk group (Figures 4C,D). In addition, we screened differentially expressed genes (DEGs) via DESeq2 and edgeR algorithms. As shown in Figures 4E–G, 162 DEGs were identified. KEGG pathway enrichment analysis proved that the DEGs primarily enriched in tumor-immune related signaling pathways including TNF signaling pathway, JAK-STAT signaling pathway, and IL-17 signaling pathway (Figure 4H). These findings suggested that the risk model consisted of hub m6A regulators and played a vital role through mediating tumor immunity.

### Prognostic Role of TIICs and Correlation With Hub m6A Regulators

**FIGURE 5:**
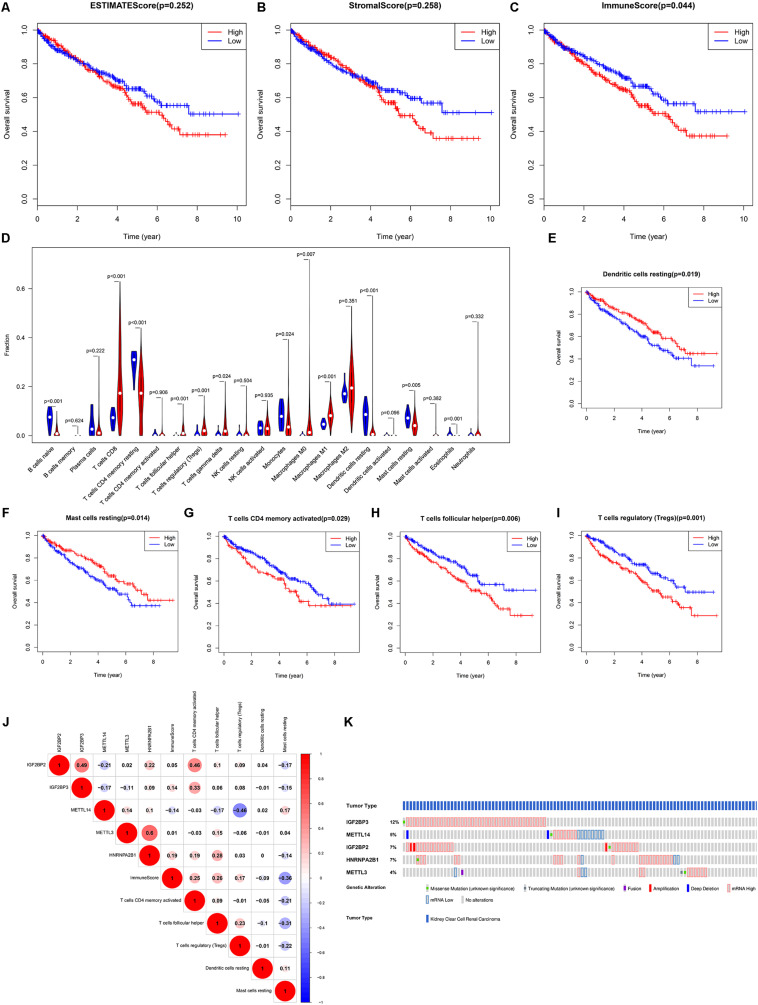
The prognostic role of TIICs and the correlation between TIICs and five hub m6A regulators. **(A–C)** ESTIMATE analysis indicated that only the immune score had good prognostic value and patients with a high immune score had poor OS. **(D)** Identification of differentially infiltrated TIICs between ccRCC tissues and normal tissues via CIBERSORT algorithm. Further survival analysis revealed that high infiltration of resting dendritic cells **(E)**/resting mast cells **(F)** predicted better OS and high infiltration of activated memory CD4 T cells **(G)**/helper follicular T cells **(H)**/regulatory T cells **(I)** predicted poor OS. **(J)** The correlation between hub m6A regulators and hub TIICs. **(K)** The genetic alteration of hub m6A regulators. TIICs, tumor-infiltrating immune cells; ESTIMATE, Estimation of STromal and Immune cells in Malignant Tumor tissues using Expression data; OS, overall survival; ccRCC, clear cell renal cell carcinoma.

According to the previous analysis, we further focused on the role of TIICs in ccRCC. ESTIMATE was performed to calculate the purity of immune cells and stromal cells. Survival analysis indicated that a high ImmuneScore predicted poor OS (Figures 5A–C). Therefore, CIBERSORT was utilized to analyze the distribution of 22 TIICs. As shown in Figure 5D, there were twelve differential TIICs between ccRCC samples and normal samples. Further survival analysis revealed that high infiltration of resting dendritic cells/resting mast cells and low infiltration of activated CD4 memory T cells/follicular helper T cells/regulatory T cells predicted shorter OS in ccRCC (Figures 5E–I), which were regarded as hub TIICs. Correlation analysis exhibited that IGF2BP2 was significantly positively associated with activated CD4 memory T cells (R = 0.46) and METTL14 was significantly negatively associated with regulatory T cells (Tregs) (R = −0.46) (Figure 5J). Thus, we inferred that IGF2BP2 might promote the infiltration of activated CD4 memory T cells and METTL14 might decrease the infiltration of Tregs in ccRCC tissues. Moreover, we found that the five hub m6A regulators all exhibited genetic alterations in ccRCC (Figure 5K). Taken together, IGF2BP2 and METTL14 were selected for subsequent analysis.

### METTL14 Was Down-Regulated and Closely Associated With Clinical Traits in ccRCC

**FIGURE 6:**
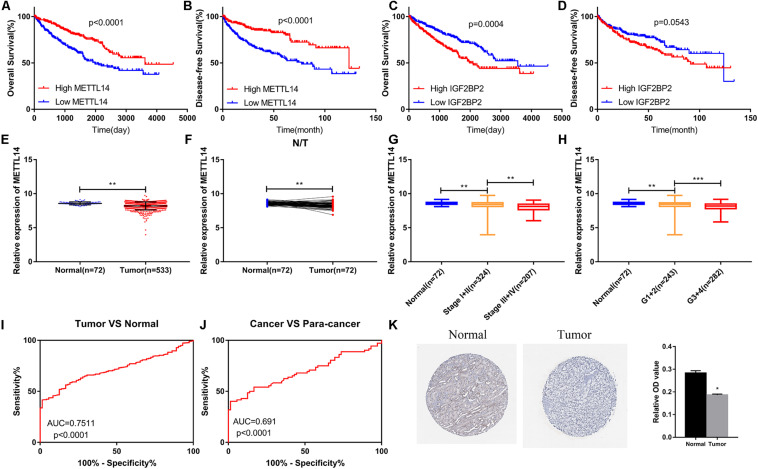
The METTL14 expression level was closely associated with various clinical traits in ccRCC. **(A,B)** High expression of METTL14 predicted better OS and DFS. **(C,D)** High expression of IGF2BP2 predicted poor OS and did not affect DFS. **(E,F)** METTL14 was down-regulated in ccRCC samples. **(G,H)** The expression level of METTL14 was significantly negatively correlated to TNM stage and Grade. **(K)** IHC result from the HPA database verified that the expression of METTL14 was decreasing in ccRCC. ccRCC, clear cell renal cell carcinoma; OS, overall survival; DFS, disease-free survival; TNM, Tumor Node Metastasis; IHC, immunohistochemistry; HPA, The Human Protein Atlas. **(I,J)** Expression of METTL14 differentiates the adjacent normal tissues and ccRCC cancer tissues. *P < 0.05; **P < 0.01; ***P < 0.001.

Survival analysis indicated that the high expression of METTL14 predicted better OS (Figure 6A) and disease-free survival (DFS) (Figure 6B). Patients with high expression of IGF2BP2 had shorter OS (Figure 6C), but DFS analysis of IGF2BP2 had no statistical difference (Figure 6D). Therefore, METTL14 was selected as the key gene for further study. In the TCGA KIRC dataset, the expression of METTL14 decreased in ccRCC tissues (Figures 6E,F). As shown in Figures 6G,H, the expression of METTL14 was negatively correlated with the TNM stage and Grade. The ROC curve demonstrated that the expression of METTL14 could effectively differentiate ccRCC tissues from normal tissues (Figures 6I,J). In the HPA database, we also found that the protein expression level of METTL14 was down-regulated in ccRCC tissues (Figure 6K).

### METTL14 Might Inhibit the Infiltration of Tregs via Suppressing CCL5

**FIGURE 7:**
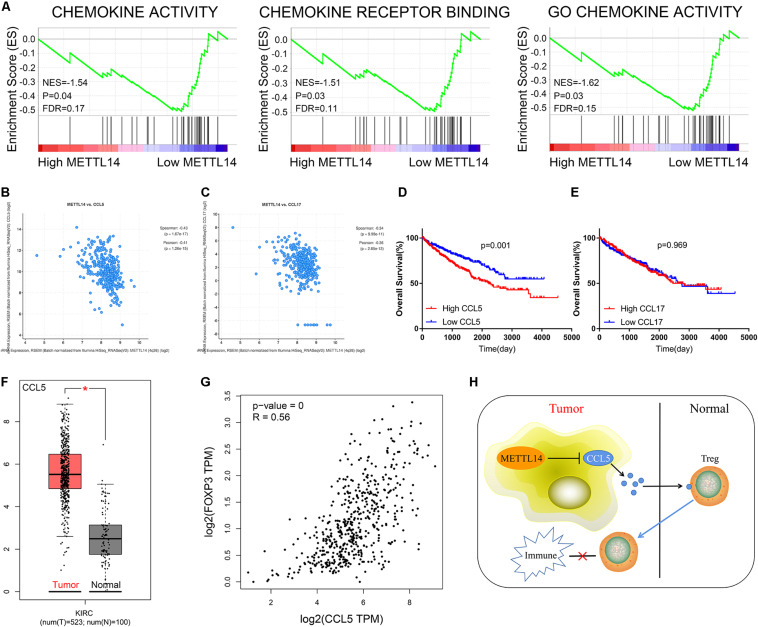
CCL5 was a potential downstream targeted molecule of METTL14. **(A)** GSEA indicated that low expression of METTL14 was enriched in chemokine-related pathways. Further analysis uncovered that METTL14 was significantly negatively associated with CCL5 **(B)** and CCL17 **(C)**. **(D)** Survival analysis exhibited that patients with high expression of CCL5 had poor OS. **(E)** The expression of CCL17 did not affect OS. **(F)** CCL5 was elevated in ccRCC tissues. **(G)** The expression of CCL5 was markedly positively correlated to FOXP3 in ccRCC. **(H)** The schematic representation of the potential molecular mechanism of METTL14/CCL5/Tregs axis in ccRCC. GSEA, gene set enrichment analysis; OS, overall survival; ccRCC, clear cell renal cell carcinoma. *P < 0.05; **P < 0.01; ***P < 0.001.

GSEA was performed to analyze the specific molecular mechanisms of METTL14 in ccRCC. The results indicated that the low expression of METTL14 was mainly enriched in chemokine related pathways (Figure 7A). We then analyzed the correlation between METTL14 and members of the CCL/CXCL superfamily. According to the threshold of correlation, CCL5 (Figure 7B, Spearman = −0.43, Pearson = −0.41) and CCL17 (Figure 7C, Spearman = −0.34, Pearson = −0.36) were regarded as the potential downstream targets of METTL14 (Table 2). Survival analysis showed that only CCL5 had a good prognostic value and high CCL5 expression predicted poor OS (Figures 7D,E). These results suggest that CCL5 might play a more important role than CCL17 in ccRCC. Moreover, CCL5 was up-regulated in ccRCC (Figure 7F) and positively correlated to FOXP3 (Figure 7G). Therefore, we speculate that the METTL14/CCL5/Tregs axis might be a potential pivotal regulated pathway of tumor immunity and progression in ccRCC (Figure 7H), a subject worthy of further in-depth study.

**TABLE 2:** The correlation between METTL14 and chemokines in ccRCC.

Correlated gene	Spearman’s correlation	Pearson’s correlation	*p*-value	q-value
CCL5	−0.43	−0.41	1.67E-17	1.02E-16
CCL17	−0.34	−0.36	9.98E-11	3.61E-10
CXCL13	−0.34	−0.27	3.69E-11	1.38E-10
CXCL1	−0.28	−0.26	7.34E-08	2.07E-07
CXCL16	−0.28	−-0.20	1.06E-07	2.96E-07
CCL19	−0.28	−0.29	1.07E-07	2.99E-07
CCL26	−0.27	−0.34	1.64E-07	4.48E-07
CCL25	−0.27	−0.22	4.35E-07	1.15E-06
CCL20	−0.23	−0.21	1.69E-05	3.81E-05
CCL11	−0.22	−0.23	2.77E-05	6.11E-05
CXCL2	−0.22	−0.02	2.79E-05	6.14E-05
CXCL5	−0.21	−0.17	5.42E-05	1.16E-04
CXCL6	−0.20	−0.14	1.65E-04	3.32E-04
CCL15	−0.20	−0.11	1.93E-04	3.86E-04
CCL21	−0.19	−0.19	2.35E-04	4.66E-04
CXCL14	−0.19	−0.14	3.60E-04	7.00E-04
CCL23	−0.19	−0.17	4.49E-04	8.63E-04
CXCL4	−0.18	−0.14	7.51E-04	1.41E-03
CCL4	−0.17	−0.13	1.23E-03	2.24E-03
CCL3	−0.16	−0.16	2.04E-03	3.60E-03
CXCL3	−0.13	−0.07	1.72E-02	2.65E-02
CXCL8	−0.12	−0.05	2.64E-02	3.93E-02
CCL22	−0.10	−0.06	5.80E-02	8.14E-02
CCL18	−0.10	−0.08	6.59E-02	9.14E-02
CCL13	−0.09	−0.10	7.95E-02	1.08E-01
CCL1	−0.09	−0.11	8.17E-02	1.11E-01
CCL2	−0.09	−0.07	9.71E-02	1.30E-01
CCL27	−0.05	−0.03	3.10E-01	3.68E-01
CCL7	−0.05	0.00	3.36E-01	3.96E-01
CXCL7	−0.05	0.07	4.00E-01	4.61E-01
CCL8	−0.04	−0.06	4.51E-01	5.13E-01
CCL14	−0.04	−0.10	4.88E-01	5.50E-01
CXCL17	−0.04	−0.10	4.90E-01	5.51E-01
CXCL9	−0.02	0.03	6.94E-01	7.40E-01
CCL16	0.01	0.06	8.38E-01	8.66E-01
CCL28	0.04	0.01	4.98E-01	5.59E-01
CXCL12	0.06	0.07	2.75E-01	3.30E-01
CXCL11	0.07	0.13	1.88E-01	2.36E-01
CXCL10	0.07	0.13	1.82E-01	2.28E-01
CCL24	0.10	0.16	5.74E-02	8.07E-02

*ccRCC, clear cell renal cell carcinoma.*

## Discussion

ccRCC is a common lethal tumor that is characterized as involving high morbidity and mortality. For advanced and metastatic ccRCC, molecularly targeted therapy (Cowey and Hutson, 2010) was selected as the first-line treatment, which improves the prognosis of patients. In recent years, immunotherapy has become the latest research hotspot. A lot of immunotherapeutic agents that target cytokine or checkpoint pathways are designed to enhance the response of the immune system in attacking tumor cells (Carosella et al., 2015; Deleuze et al., 2020). However, there are still some patients with medicine resistance and poor survival. Therefore, it is necessary to further clarify the molecular mechanisms of tumor immunity and find novel effective therapeutic targets in ccRCC.

This study analyzed the potential diagnostic and prognostic value of m6A regulators. Combined with TIICs analysis, we found that the METTL14/CCL5/Tregs axis was a potential tumor immune regulative pathway in ccRCC.

Recently, increasing studies have reported that m6A modification of RNA, which is mediated by three type regulators (“writer,” “eraser,” and “reader”), and is involved in central events in biology (Meyer and Jaffrey, 2014; Liu and Pan, 2016). METTL14 is a member of “writer” and identified as a catalytic component of the methyltransferase complex responsible for creating m6A modifications in various biological processes (Huang et al., 2019). Animal experiments verified that METTL14 could regulate striatal function in a mouse model (Koranda et al., 2018). In the hematological system, METTL14 suppressed hematopoietic stem/progenitor differentiation and promoted leukemogenesis via mediating MYB/MYC (Weng et al., 2017). Moreover, Chen et al. revealed that METTL14 inhibited colorectal cancer growth and metastasis through targeting miR-375. Our study uncovered that METTL14 was down-regulated in ccRCC and significantly negatively correlated to tumor stage. Survival analysis also demonstrated that the high expression of METTL14 predicted better OS and DFS.

We still lack studies of the correlation between METTL14 and tumor immunity. In this study, we indicated that METTL14 might act as a modulator of Tregs to regulate tumor immunity in ccRCC. Previous studies have reported that immunosuppressive cells including regulatory T (Treg) (Sakaguchi, 2000) cells, myeloid derived suppressor cells (MDSCs) (Gabrilovich, 2017), and tumor-associated macrophages (TAMs) (Mantovani et al., 2017) inhibit effective anti-tumor immune responses. According to molecular markers, Tregs fall into two major categories: resting/naïve Treg (rTreg, FOXP3^*lo*^CD45RA^+^CD25^*lo*^) and effector/activated Treg (eTreg, FOXP3^*hi*^CD45RA^–^CD25^*hi*^) (Ohue and Nishikawa, 2019). Effector/activated Treg has a strong immunosuppressive function and inhibits anti-tumor immunity through various molecular mechanisms, such as the regulation of the immune checkpoint molecules (Kamada et al., 2019), induction of apoptosis for immune effector cells (Gotot et al., 2018), and production of immunosuppressive cytokines (Collison et al., 2007). It has been reported that inhibition of Nr4a suppresses tumor progression via weakening Treg-mediated immune tolerance (Hibino et al., 2018). Our study also demonstrated that infiltration of Tregs was a potential risk factor in ccRCC.

Previous studies have also revealed that Tregs accumulate in the tumor microenvironment (TME) through chemokine gradients (Bromley et al., 2008). CCR4-CCL17, CCR8-CCL1, and CXCR3-CXCL9/10/11 are the major signaling pathways for Tregs chemotaxis (Ohue and Nishikawa, 2019). Wang et al. verified that tumor-derived FOXP3 recruited FOXP3(+)Treg cells by activating CCL5 in pancreatic ductal adenocarcinoma (Wang et al., 2016). Analogously, You et al. also revealed that ovarian cancer stem cells recruited Tregs via the CCL5-CCR5 axis (You et al., 2017). In this study, GSEA and correlation analysis indicated that CCL5 was negatively associated with METTL14, which inferred that CCL5 was a potential target for METTL14. CCL5 (C-C Motif Chemokine Ligand 5), also named RANTES, can activate several chemokine receptors including CCR1, CCR3, CCR4, and CCR5 for chemotaxis of immune cells. Our research also indicated that the expression of CCL5 was positively correlated to FOXP3 in ccRCC tissue.

## Conclusion

In conclusion, METTL14 was identified as a key m6A RNA methylation regulator via integrated bioinformatics analysis. Combining this approach with tumor immune analysis, we found that METTL14 might suppress tumor progression through mediating Tregs. Further study indicates that CCL5 served as a potential downstream molecule of METTL14 to regulate Tregs chemotaxis. Taken together, these findings suggest that the METTL14/CCL5/Tregs axis is expected to be a novel therapeutic target for ccRCC. However, the specific mechanisms require further research.

## Data Availability Statement

Publicly available datasets were analyzed in this study. This data can be found here: https://www.cancer.gov/tcga.

## Ethics Statement

We certify that this manuscript is original and has not been published and will not be submitted elsewhere for publication. No data have been fabricated or manipulated (including images) to support our conclusions.

## Author Contributions

XZ and KC designed this study. TX and SG performed data collection and analysis, wrote the manuscript, and contributed to preparing and making figures and tables. TX, SG, HR, and JL performed the majority of the experiments. YL, DL, and JT collected the clinical samples and managed the clinical data. JT and JS reviewed the relevant literature. HY, XZ, and KC provided conceptual advice and critically reviewed the manuscript. All authors read and approved the final manuscript.

## Conflict of Interest

The authors declare that the research was conducted in the absence of any commercial or financial relationships that could be construed as a potential conflict of interest.

## Footnotes

1 https://seer.cancer.gov/

2 https://www.cancer.gov/tcga

3 https://CRAN.R-project.org/package=corrplot

4 https://string-db.org

5 https://CRAN.R-project.org/package=ggplot2

6 https://CRAN.R-project.org/package=survival

7 https://cibersort.stanford.edu/

## Supplementary Material

Supplementary Figure 1 Flow chart of study design. The processes of data analysis are shown in the flow chart. TCGA, The Cancer Genome Atlas; KIRC, Kidney Clear Cell Carcinoma; DE-m6A, differentially expressed m6A; ESTIMATE, Estimation of STromal and Immune cells in Malignant Tumor tissues using Expression data; TIICs, tumor-infiltrating immune cells; LASSO, least absolute shrinkage and selection operator; GSEA, gene set enrichment analysis; GSVA, gene set variation analysis; OS, overall survival; DFS, disease-free survival; HPA, The Human Protein Atlas; ROC, receiver operating characteristic.
