# Supplementary figures and images for: METTL14 Acts as a Potential Regulator of Tumor Immune and Progression in Clear Cell Renal Cell Carcinoma
Source: Front Genet. 2021 May 28;12:609174. doi: 10.3389/fgene.2021.609174 (PMC8194313; doi:10.3389/fgene.2021.609174)

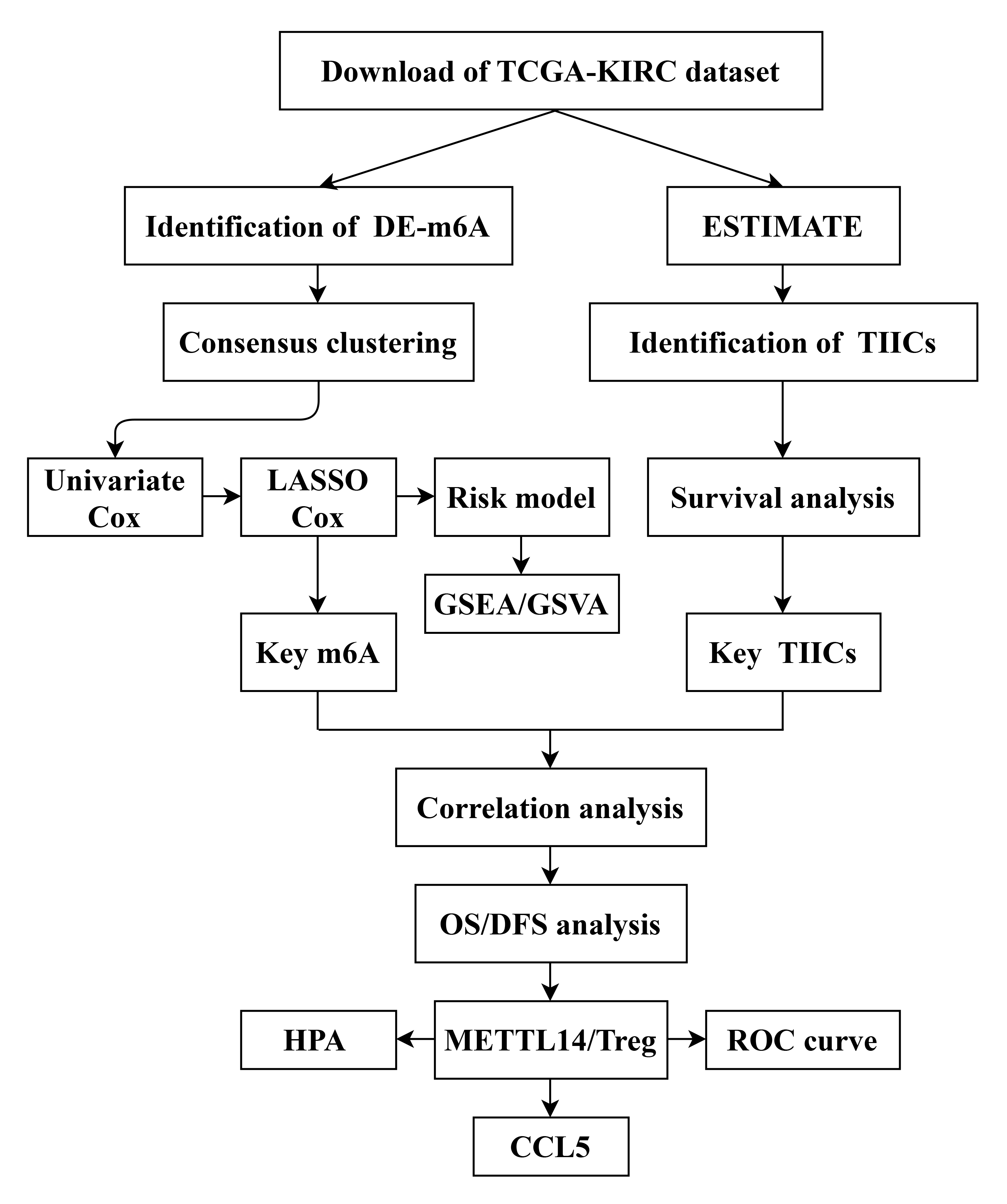

Supplement: Supplementary Figure 1 — Flow chart of study design. The processes of data analysis are shown in the flow chart. TCGA, The Cancer Genome Atlas; KIRC, Kidney Clear Cell Carcinoma; DE-m6A, differentially expressed m6A; ESTIMATE, Estimation of STromal and Immune cells in Malignant Tumor tissues using Expression data; TIICs, tumor-infiltrating immune cells; LASSO, least absolute shrinkage and selection operator; GSEA, gene set enrichment analysis; GSVA, gene set variation analysis; OS, overall survival; DFS, disease-free survival; HPA, The Human Protein Atlas; ROC, receiver operating characteristic. [file Image_1.TIF]
